# The Effect of Diabetic Neuropathy on the Frequency of Joint Replacement in the Rio Grande Valley

**DOI:** 10.7759/cureus.71652

**Published:** 2024-10-16

**Authors:** Blake Martin, Jared Hensley, Kristina Vatcheva, Kelsey Baker

**Affiliations:** 1 School of Medicine, The University of Texas Rio Grande Valley, Edinburg, USA; 2 School of Medicine, University of Texas Rio Grande Valley School of Medicine, Edinburg, USA; 3 Biostatistics and Epidemiology, University of Texas Rio Grande Valley School of Medicine, Edinburg, USA; 4 Neuroscience, The University of Texas Rio Grande Valley, Edinburg, USA

**Keywords:** arthroplasty, bone health, diabetes, diabetic neuropathy, joint, joint replacement

## Abstract

Background

Research has suggested that bone health may be impaired in patients with diabetic neuropathy, with a potential increased risk of fracture. Here, we sought to evaluate the frequency of joint replacements in individuals with and without DN. Our work may allow for prophylaxis to prevent further deterioration in bone health and reduce the necessity of surgical procedures.

Methods

We conducted a retrospective chart review, from 2019 through 2023, using the UTHealth Rio Grande Valley electronic medical records system. We evaluated odds ratios of fracture history in patients with DN, other diabetic complications, or diabetes (n=10,416). Multiple confounders were considered from patient characteristics, including age, sex, ethnicity, and county of residence. Statistical tests were performed with a significance level of 0.05 to further evaluate our data.

Results

We observed that there was no significant difference in the distribution of joint replacement across patients with DN, other diabetic complications, or diabetes. Ethnicity and sex were found to not significantly affect the odds of joint replacement. However, older individuals were significantly more likely to have joint replacement than younger individuals (p=0.0004).

Conclusion

Our results suggest that joint replacement frequency was not increased in diabetic populations in the Rio Grande Valley. Thus, we suggest that interventions to promote bone health should not be limited to specific diabetic patient groups. Further studies should be conducted to determine the exact mechanisms by which bone is affected by DN to allow for improved knowledge and care of bone health in DN patients.

## Introduction

Approximately 10.5% of the adult population worldwide (aged 20-79) has been diagnosed with diabetes [1]. It is projected that by 2045, 1 in 8 adults, approximately 783 million, will be living with diabetes, an increase of 46% [1]. One common effect of diabetes is the development of diabetic peripheral neuropathy. Diabetic peripheral neuropathy is defined as the presence of symptoms and/or signs of peripheral nerve dysfunction in subjects with diabetes mellitus (DM), after the exclusion of other causes [2]. Estimates of the prevalence of diabetic peripheral neuropathy show considerable variation, from 2.4% up to 75.1%, depending on sample selection and applied diagnostic criteria, with a suggested overall prevalence of 40.3% [2]. DN has been attributed to poor glucose control and lifestyle factors such as poor diet and lack of exercise [3]. Nerve damage in DM has also been associated with hypertension, high cholesterol, advanced kidney disease, increased weight, alcohol, and smoking [3]. Such nerve damage that accompanies DN has also been suggested to impact bone health outcomes.

Poor bone health is already increasingly recognized as a complication of type 2 diabetes mellitus (T2DM) [4]. The pathophysiological mechanisms are complex and contentious, with obesity and insulin resistance observed to have varied effects on bone, oxidative stress, and microvascular disease [4]. Intriguingly though, a recent meta-analysis found that T2DM was significantly associated with increased risk of hip and vertebral fractures [4]. Of particular interest, the greatest elevation of fracture risk in T2DM was seen at the foot, where relative risk was 37% higher than comparator populations without T2DM [4]. Authors suggested that DN, which commonly presents in the foot, may have had a potential involvement in fracture predisposition [4]. DN can decrease peripheral sensation (sensory neuropathy), impair motor coordination (motor neuropathy), and increase postural hypotension (autonomic neuropathy) [5]. Together, these can impair overall balance and increase the risk of falls and fractures [5]. In addition, the peripheral nervous system has the potential to regulate bone metabolism directly through the action of local neurotransmitters on bone cells and indirectly through the neuroregulation of the skeletal vascular supply [5].

A compelling association is emerging between DN and bone disease, but evidence is still forthcoming regarding the direct effects of DN on bone or the utility of DN as a predictor of skeletal fragility and fracture [5]. Thus, here we sought to determine if DN affects the frequency of joint replacement. Our study focused on populations in the Rio Grande Valley. The Rio Grande Valley represented a unique population to evaluate this premise since it has approximately a 2X higher incidence of DM than the nation [6]. We hypothesized that in a diabetic population, individuals with DN will have an increased frequency of joint replacement compared to individuals without DN.

This article was previously presented as a meeting abstract at the Texas A&M 18th Annual Diabetes Conference on July 12, 2024.

## Materials and methods

This study was conducted via a retrospective chart review, from January 1, 2019, through the end of December 2023. Data were extracted using the UTHealth Rio Grande Valley (RGV) electronic medical records system, focusing on the tenth revision of the International Classification of Diseases (ICD-10) codes related to DN and joint replacements. ICD-10 codes for DN were E11.40, E10.40, E11.42, E11.610, E13.42, E11.43, E11.41, andE10.43. The ICD-10 code used for joint replacement was Z47.1. Confounding variables, such as comorbidities, medication use, and physical activity levels, were identified and adjusted for in the statistical analysis to ensure robust findings. Individuals aged 18 through 95 were included while individuals outside this range were excluded. The records were analyzed by evaluating individuals’ charts with DN, other diabetic complications, or diabetes (type 1 or 2). Our evaluation groups included individuals with DN, individuals with DN who later had a joint replacement, individuals with complications other than neuropathy, individuals with complications other than neuropathy who later had a joint replacement, individuals with diabetes without complications, and individuals with diabetes without complications who later had a joint replacement. Multiple patient characteristics were also evaluated for comparison including age, sex, ethnicity/race, county of residence, body mass index (BMI), weight, and height. 

Patients’ characteristics were described by joint replacement status using mean and standard deviation (std) for continuous variables and frequency (n) and percentage (%) for categorical variables. The distributions of continuous and categorical variables for the joint replacement and non-joint replacement groups were compared using the student’s test and chi-squared test or Fisher exact test, respectively. Univariable and multiple logistic regression models were used to evaluate the association between DM complications and joint replacement. Crude and adjusted odds ratios (ORs) and their respective 95% confidence intervals (CIs) for joint replacement were estimated. Multicollinearity between the predictors in the models was assessed using variance inflation factors. Potential two-way interactions were assessed by testing, one at a time, for non-zero regression coefficients at significance level α=0.05 of the arithmetic products of all pairs of variables included in the models. The area under the receiver operating characteristic (ROC) curve, which provides a measure of the model’s ability to discriminate between those subjects who have had joint replacement versus those who have not, was calculated. The Hosmer and Lemeshow model’s goodness-of-fit test was conducted. All statistical tests were two-sided and performed at a significance level of 0.05. The significance level of 0.05 indicates that there is a 5% chance that the observed differences are due to random variation rather than actual differences between groups. Findings with p-values less than 0.05 were considered statistically significant. All statistical analyses were conducted using SAS 9.4 (SAS Institute, Inc., Cary, North Carolina, US).

## Results

Characteristics of the study population

Table 1 provides the main socio-demographic patients’ characteristics. In total 10,416 DM patients were analyzed. The county of residence could not be analyzed due to minimal available data. Only 20 (0.2%) of the diabetes patients had joint replacement and 6 (30%) of them had neuropathic complications. Forty-eight point two (48.2%) of the patients were males and an equal proportion of males and females had joint replacement (p=0.8709). The mean age of the study population was 56.8 years (std=13.8 years), and participants with joint replacement were older than participants with no joint replacement (p<0.0001). The majority of the patients who reported their ethnicity were Hispanic or Latino (80.8%) and 13 of them were with joint replacement (p<0.0001).

**Table 1:** Patient's characteristics stratified by joint replacement status * Fisher's exact test was conducted excluding patients with unreported ethnicity on a sample with size n=9172.

Characteristic	All (n=10416)	Joint replacement (n=20)	No joint replacement (n=10396)	P-value
	n (%)	n (%)	n (%)	
Age (years), mean (std)	56.8 (13.80)	68.1 (8.33)	56.8 (13.80)	<0.0001
Sex				
Male	5019 (48.2)	10 (50.0)	5009 (48.2)	0.8709
Female	5397 (51.8)	10 (50.0)	5378 (51.8)	
Ethnicity, n=9172				
Hispanic or Latino	8412 (80.8)	13 (65.0)	8399 (80.8)	0.3565*
Not Hispanic or Latino	760 (7.3)	2 (10.0)	758 (7.3)	
Declined	1244 (11.9)	5 (25.0)	51239 (11.9)	
Diabetes mellitus				
Neuropathy complications	3596 (34.5)	6 (30.0)	3590 (34.5)	0.5651
Complications excluding neuropathy	3298 (31.7)	5 (25.0)	3293 (31.7)	
No complications	3522 (33.8)	9 (45.0)	3513 (33.8)	

Factors associated with joint replacement in the study population

Based on univariable and multivariable logistic regression analyses (Table 2), age was the only variable significantly associated with the log odds of joint replacement. For every one-year increase in age, the odds of joint replacement in diabetes patients increased by 6% (OR=1.06, 95% CI: 1.03, 1.10; p=0.0004), adjusting for the effect of sex and diabetes complications (Table 2). Sex was not significantly associated with the log odds of joint replacement. Ethnicity was not statistically analyzed in regard to joint replacement due to the small sample size.

**Table 2:** Crude and model-based adjusted associations with fraction based on logistic regression, n=10416 * Adjusted OR were estimated using all data (n=10416) based on multiple logistic regression models including age, sex, and diabetes complications.

Characteristic	Crude OR (95% CI)	P-value	Adjusted OR (95% CI)*	P-value
Age (years)	1.06 (1.03, 1.10)	0.0003	1.06 (1.03, 1.10)	0.0004
Sex				
Male	1.08 (0.25, 2.59)	0.8709	1.09 (0.45, 2.63)	0.8458
Female	reference			
Ethnicity, n=9172				
Hispanic or Latino	0.59 (0.13, 2.60)	0.4830	n/a	n/a
Not Hispanic or Latino	reference		n/a	n/a
Diabetes mellitus				
Neuropathy complications	0.65 (0.23, 1.84)	0.4182	0.65 (0.23, 1.85)	0.4273
Complications excluding neuropathy	0.59 (0.20, 1.77)	0.3488	0.61 (0.20, 1.83)	0.3768
No complications	reference		reference	

Overall, this study found that there was no significant difference in the distribution of joint replacement across the different diabetic groups analyzed. Thus, joint replacement frequency was similar among individuals with diabetes and neuropathy, individuals with diabetes and other complications, and individuals with diabetes and no complications. Ethnicity and sex were not significantly associated with the odds of joint replacement. However, older individuals were significantly more likely to have joint replacement than younger individuals.

## Discussion

There is no previous literature on the effect of DN on the frequency of joint replacement. However, recent literature has suggested that DN may have negative effects on bone health, which may lead to an increase in joint replacement in this population [5]. Although we expected increased joint replacement due to these new findings, our study did not find any increased frequency of joint replacement. Our observed lack of significant difference in joint replacement frequency among the different diabetic groups, and overall reduced joint replacement prevalence, may be due to the patient population in the RGV. Indeed, our data showed that individuals receiving a joint replacement more frequently identified as Latino/Hispanic (Table 1). The population residing in the Rio Grande Valley region is largely Mexican-American and of low socioeconomic status, indicating that individuals may not be as financially stable when compared to other areas of the United States [7]. Also, many individuals in this area lack insurance, which may prevent joint replacement and other elective surgeries [8,9]. Thus, due to financial instability and lack of insurance, individuals may have not elected to have joint replacement surgery.

This study did not show a significant difference in the prevalence of joint replacement in men compared to women, a finding that does not agree with previous literature [10]. We believe the construct of machismo may also have impacted the low number of joint replacements of males seen in this study. The machismo construct originated from work with Mexican-origin men and has been used colloquially to characterize all Latino men [11]. Conventional beliefs about masculinity, masculine identity, and gender expression norms, are socially constructed and largely influence an individual’s perception and behavior regarding what they think it means to be a man, particularly in individuals of Latino heritage [11]. Men’s behaviors in the machismo construct are shaped by trying to explicitly avoid behaviors that are perceived as feminine such as eating healthy, applying sunscreen, and possibly avoiding seeking help for medical conditions [11].

Our study has several limitations. First, since our study is a retrospective analysis, the study design limits the ability to establish causality. Additionally, the reliance on electronic medical records may result in underreporting of certain complications due to incomplete data entries. Finally, we acknowledge that the results found in the RGV population analyzed in this study may not be completely generalizable to the general population as there are many differences in the population in South Texas, such as a dominant Hispanic population, large number of immigrants, and large number of individuals without insurance coverage.

## Conclusions

We found that no significant difference in the proportion of joint replacement existed between the following groups: Individuals with diabetes and only neuropathy as a complication, individuals with diabetes and complications other than neuropathy, and individuals with diabetes and no complications. According to these results, interventions to promote bone health and prevent joint arthroplasty should not be limited to specific diabetic patient groups but should be considered for all diabetic patients, and further studies should be conducted to determine the exact mechanisms by which bone is affected by DN to allow for improved knowledge and care of bone health in DN patients to prevent possible future joint arthroplasty.
